# MPPa-PDT suppresses breast tumor migration/invasion by inhibiting Akt-NF-κB-dependent MMP-9 expression via ROS

**DOI:** 10.1186/s12885-019-6374-x

**Published:** 2019-11-29

**Authors:** Liyi Huang, Haidan Lin, Qing Chen, Lehua Yu, Dingqun Bai

**Affiliations:** 1 Department of Rehabilitation Medicine, The First Affiliated Hospital of Chongqing Medical University, Chongqing, 400016 People’s Republic of China; 2 Department of Rehabilitation Medicine, The Second Affiliated Hospital of Chongqing Medical University, Chongqing, 400010 People’s Republic of China

**Keywords:** Photodynamic therapy, Reactive oxygen species, Breast tumor, Migration, Invasion

## Abstract

**Background:**

Breast cancer is one of the most commonly diagnosed cancers in women, with high morbidity and mortality. Tumor metastasis is implicated in most breast cancer deaths; thus, inhibiting metastasis may provide a therapeutic direction for breast cancer. In the present study, pyropheophorbide-α methyl ester-mediated photodynamic therapy (MPPa-PDT) was used to inhibit metastasis in MCF-7 breast cancer cells.

**Methods:**

Uptake of MPPa was detected by fluorescence microscopy. Cell viability was evaluated by the Cell Counting Kit-8 (CCK-8). ROS generation was detected by 2′,7′-dichlorodihydrofluorescein diacetate (DCFH-DA). The migration of cells was assessed by wound healing assay, and invasion ability was assessed by Matrigel invasion assay. Levels of MMP2 and MMP9 were measured by PCR. Akt, phospho-Akt (Ser473), phospho-NF-κB p65 (Ser536) and NF-κB p65 were measured by western blotting. The F-actin cytoskeleton was observed by immunofluorescence. Lung tissue was visualized by hematoxylin and eosin staining.

**Results:**

Following MPPa-PDT, migration and invasion were decreased in the MCF-7 cells. MPPa-PDT downregulated the expression of MMP2 and MMP9, which are responsible for the initiation of metastasis. MPPa-PDT reduced the phosphorylation of Akt and NF-κB. MPPa-PDT also reduced the expression of F-actin in cytoskeleton in MCF-7 cells. These effects were blocked by the reactive oxygen species scavenger NAC or the Akt activator SC79, while the PI3K inhibitor LY294002 or the Akt inhibitor triciribine enhanced these effects. Moreover, MPPa-PDT inhibited tumor metastasis and destroyed F-actin in vivo.

**Conclusion:**

Taken together, these results demonstrate that MPPa-PDT inhibits the metastasis of MCF-7 cells both in vitro and in vivo and may be involved in the Akt/NF-κB-dependent MMP-9 signaling pathway. Thus, MPPa-PDT may be a promising treatment to inhibit metastasis.

## Background

Breast cancer is the second leading cause of cancer death in women around the world [1]. Metastasis is the dominant cause of death in breast cancer patients [2]. It is related to many elements, such as destruction of the extracellular matrix (ECM) [3] and generation of new metastatic tumors in secondary sites after transport in blood and lymph vessels [4]. Matrix metalloproteinases (MMPs) play a crucial role in the degradation of the ECM and the subsequent invasion and metastasis of tumor cells [5, 6]. MMPs are zinc-dependent endopeptidases that include gelatinases, collagenases, stromelysins, and membrane-associated MMPs [1]. The relationships of MMP-2 and MMP-9 to the degradation of the ECM and tumor metastasis [7] are significant; thus, they are regarded as progression markers in breast cancer.

Phosphoinositide 3-kinase (PI3K)/protein kinase B (Akt) is an important signaling pathway that is involved in tumor cell growth, proliferation, apoptosis, metabolism, angiogenesis, metastasis and immunity [8–10]. It also has a close connection with the NF-κB signaling pathway, in which the phosphorylation of Akt can activate NF-κB [11], triggering the regulation of downstream MMP-2 and MMP-9, regulating cancer cell proliferation, migration and invasion [12, 13]. Inhibition of MMP-2 and MMP-9 [14] may be a suitable therapeutic option for cancer.

Photodynamic therapy (PDT), as a valid therapy modality for multiple solid tumors, is minimally invasive and innoxious and possesses selective cytotoxicity for targeted cells [15]. It is based on photosensitizers and laser light with a specific wavelength, causing the production of reactive oxygen species (ROS) and inducing tumor cell apoptosis/necrosis [16]. PDT has been clinically used for various cancers, including cervical [17], lung [18], bladder [19], skin [20] and head and neck cancers [5, 21].

Pyropheophorbide-α methyl ester (MPPa), a derivate of chlorophyll [22], provides more advantages due to its better absorbance of and stronger permeability to PDT compared with first-generation photosensitizers. Our previous studies demonstrated that MPPa-PDT can inhibit breast cancer cell growth [23]; however, the role of MPPa-PDT on invasion and migration in breast cancer is not clear. In the present study, we observed that MPPa-PDT inhibited breast cancer cell MCF-7 metastasis and the underlying molecular mechanisms, which may provide important implications for breast cancer treatment.

## Methods

### Major reagents

Pyropheophorbide α methyl ester (MPPa, C34H36N4O3) was obtained from Sigma-Aldrich (St. Louis, MO). The laser (630 nm) was purchased from Chongqing Jingyu Laser Technology Co., Ltd. (Chongqing, China). Dulbecco’s modified Eagle’s medium (DMEM) was obtained from HyClone (Logan, UT). The Cell Counting Kit-8 (CCK-8) was procured from Dojindo Molecular Technologies (Kumamoto, Japan). Akt (catalogue number: 4691, dilution: 1:1000), phospho-Akt (catalogue number: 4060, Ser473, dilution: 1:2000), phospho-NF-κB p65 (catalogue number: 3033, Ser536, dilution: 1:1000) and NF-κB p65 (catalogue number: 8242, dilution: 1:1000) were obtained from Cell Signaling Technology (Danvers, MA). GAPDH was purchased from Sungene Biotech (Tianjin, China). Loading control was obtained from Beyotime (Shanghai, China). Trypsin and Actin-tracker Green were procured from Beyotime (Shanghai, China).

### Cell culture

The human breast cell line MCF-7 (Shanghai Institute of Cell Biology China, catalogue number: SCSP-531) was cultured in DMEM supplemented with 10% fetal bovine serum and 1% penicillin and streptomycin (Beyotime, Shanghai, China). MCF-7 cells were routinely cultured in 5% CO_2_ at 37 °C.

### PDT protocol

Four groups were designed in the present study—A: control group; B: MPPa-only group; C: laser-only group; D: MPPa-PDT group. After cell attachment, the media of groups A and C were replaced with fresh media, while the media of groups B and D were replaced with media containing MPPa (2 μmol/L). Cells of groups B and D were washed with PBS to remove the MPPa after 12 h, and then groups C and D were exposed to LED light (630 nm, 30 mW/cm^2^) for 30, 60, 90, 120, or 180 s to obtain an energy density of 0.9, 1.8, 2.7, 3.6, or 5.4 J/cm^2^, respectively. Energy density (J/cm^2^) = power (mW/cm^2^) × irradiation time (s). The cells were cultured in the incubator after irradiation. All the operations were performed in the dark.

### Detection of intracellular MPPa

MPPa sparkles with red fluorescence upon corresponding excitation. To observe the uptake of MPPa in MCF-7 cells, cells were seeded into 6-well plates and incubated with 2 μmol/L MPPa for different times (0 h, 3 h, 6 h, 12 h, and 24 h). The intracellular accumulation of MPPa was detected by fluorescence microscopy (Leica DMRE Fluorescence Microscope, Germany).

### Cell viability and cytotoxicity tests

Cells were seeded in 96-well plates at a density of 5 × 10^3^ cells/well. After 24 h of different treatments, the medium of each well was discarded, 100 μl serum-free medium containing 10 μl CCK-8 solution was added to each well, and the cells were further incubated for 1 h in 5% CO_2_ at 37 °C. Optical densities (ODs) were measured by a microplate reader. Cell viability (%) = (Average OD of experiment group – Average OD of blank group)/ (Average OD of control group – Average OD of blank group) × 100%. The wells of the blank group contained only DMEM with CCK-8 solution.

### ROS detection

MCF-7 cells were seeded in 24-well plates. After the above mentioned treatments, the medium of each group was replaced by DCFH-DA (Invitrogen, Paisley, UK) at a concentration of 10 μmol/L. After 30 min, the cells were washed with PBS three times to wash away the extracellular DCFH-DA. Fluorescence microscopy was used to detect the production of ROS (Zeiss Fluorescence Microscope, Germany).

### Wound healing assay

MCF-7 cells were seeded in 6-well plates at a density of 1 × 10^5^ cells/well. The MPPa-only group and MPPa-PDT group were subjected to MPPa-medium (2 μmol/ml) and incubated for 12 h. Then the medium was replaced with PBS, and a scratch was made with a sterile pipet tip (200 μl) in all groups. The detached cells were removed. The corresponding irradiation was performed in the laser-only group and MPPa-PDT group. Images were taken immediately after irradiation and 24 h later using a fluorescence microscope. The area of the scratch was analyzed using ImageJ software.

### Matrigel invasion assay

A Matrigel invasion assay was utilized to evaluate the invasiveness of cells pre- and post-PDT. Cells were divided into 4 groups and received the corresponding treatment. Matrigel (Becton Dickinson, Bedford, MA) was added into Transwell inserts (Corning Costar, Tokyo, Japan) for solidification in a 24-well plate. Cells were collected and resuspended in serum-free medium and then transferred into the upper chambers, 5 × 10^4^ cells for each Transwell insert. The lower wells were supplemented with 750 μl of complete medium. After 48 h, the cells that did not invade the lower surface of the transwell inserts were cleaned with a cotton swab, while the invaded cells of the inserts were fixed with 4% paraformaldehyde, washed with PBS three times and subjected to crystal violet staining (Beyotime, Shanghai, China). Transwell inserts were visualized by light microscopy (Zeiss Fluorescence Microscope, Germany).

### Real-time PCR analysis

RNA was extracted by RNAiso Plus reagent (Takara) at 24 h after irradiation. The compounds of cells and RNAiso Plus reagent were centrifuged for 15 min at 12000 rpm, and the supernatant was collected in new EP tubes to obtain the sediment of RNA by isopropyl alcohol precipitation. Extracted RNA was purified with 75% ethyl alcohol and suspended in 20 μl diethyl pyrocarbonate (DEPC water). The cDNAs were synthesized through reverse transcription reactions with a mixture following the instructions (Takara, Japan), and real-time polymerase-chain reaction (PCR) was performed with CFX96™ Bio-Rad. Primers were synthesized by Qingke (Chongqing, China): ATGGGGAAGGTGAAGGTCGG (Forward) and GACGGTGCCATGGAATTTGC (Reverse) for GAPDH; (Forward) CCGCTCACCTTCACTCG and CTCCGCGACACCAAACT (Reverse) for MMP9; and (Forward) CCCACTGCGGTTTTCTCGAAT and CAAAGGGGTATCCATCGCCAT (Reverse) for MMP2.

### Western blotting

To collect the cell protein at the indicated time points (times are shown in the figure) or after 24 h, cells were lysed in RIPA (Radio Immunoprecipitation Assay) Lysis buffer containing PMSF (Phenylmethylsulfonyl fluoride) (RIPA:PMSF = 100:1) and centrifuged at 12000 g at 4 °C. Protein concentrations were quantified by BCA assay and prepared with RIPA and loading buffer to make the final concentrations the same. Target proteins were separated by gel electrophoresis and then transferred to PVDF membranes (Millipore). Five percent nonfat dry milk was used to block membranes at room temperature, and Tris-buffered saline plus tween (TBST) was used to wash the membranes. Then, the membranes were exposed to homologous primary antibodies and shaken tardily at 4 °C overnight. The next day, the membranes were washed with TBST to remove antibodies in excess and then exposed to secondary antibodies of the corresponding species. The images were obtained by Fusion with the ECL coreaction (Advansta, USA). All experimental results were analyzed by Fusion (Fusion, Vilber Lourmat, France).

### F-actin cytoskeleton analysis

MCF-7 cells were seeded onto glass coverslips in a 12-well plate. After treatment, the coverslips were collected and fixed with 4% paraformaldehyde (PFA). Cells were permeabilized using 0.1% Triton X and then blocked in 5% BSA. Cells were stained with Actin-tracker Green (Beyotime Biotechnology, China) for 1 h at room temperature. Then the surface of slides was smeared with Antifade mounting medium, and all coverslips were transferred onto slides. The image series were captured using a ZEISS LSM800 confocal microscope (Carl Zeiss AG, Germany).

### Tumor models

All female nude mice (4–6 w) were obtained from the Experimental Animal Center of Chongqing Medical University. All animal studies abided by the ethics guidelines of the Animal Ethics Committee of Chongqing Medical University. The nude mice were inoculated subcutaneously with 1 × 10^6^ MCF-7 cells in 100 μL PBS to form MCF-7 xenograft breast cancer. These mice were randomly divided into four groups when the tumor grew to approximately 100 mm^3^: control group, MPPa group, laser group and MPPa-PDT group (*n* = 3 in each group). MPPa was administered intravenously to the tumor-bearing mice at a concentration of 15 mg/kg, and the laser was applied 12 h after injection at 120 J/cm^2^ every 2 days for 10 days. Tumor sizes and body weights were recorded during treatment.

$$ \mathrm{Tumor}\ \mathrm{Volume}\ \left(\mathrm{TV}\right)=\mathrm{Length}\times {\mathrm{Width}}^2/2. $$

### Tissue histology and immunohistochemistry

Nude mice were euthanized by cervical dislocation, and the major organs were harvested on day 16. Lung tissues were formalin-fixed, paraffin-embedded, and visualized by hematoxylin and eosin staining. Tumor tissues were stained by Actin-tracker Green and DAPI as well as for collagen for 30 min at room temperature for immunohistochemistry.

### Statistical analysis

Data are shown as mean ± SD. GraphPad Prism Software version 7.00 (San Diego, CA) was used for statistical analysis. The method of statistical analysis was one-way analysis of variance (ANOVA). *P* < 0.05 was regarded as statistically significant.

## Results

### MPPa-PDT suppresses MCF-7 cell viability

**Fig. 1:**
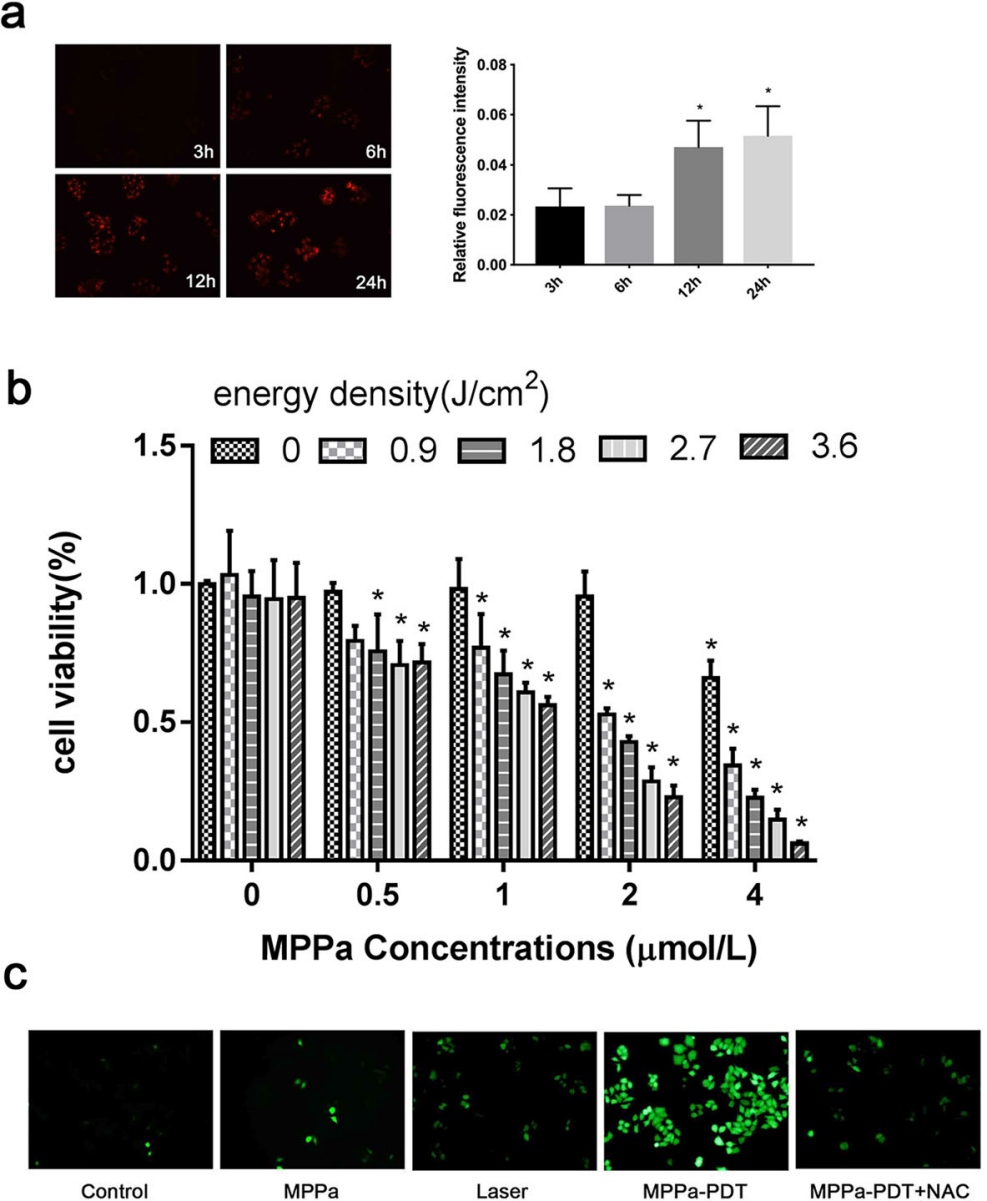
MPPa-PDT influences the cell viability of MCF-7 cells. **a** Uptake of MPPa for different times measured by fluorescence microscopy (magnification, × 200). **b** The effect of MPPa-PDT on the cell viability measured by CCK-8 assay after 24 h. **c** The effect of MPPa-PDT on ROS production detected by DCFH-DA staining (magnification, × 200). (*n* = 3; *P < 0.05 versus control group, **P < 0.01 versus control group)

As time progressed, the uptake of MPPa in MCF-7 cells increased speedily in the first 12 h, while there was no significant increase within the following hours (Fig. 1a). For this reason, 12 h was chosen for follow-up experiments. The CCK-8 assay results demonstrated that cell viability exhibited no significant difference compared with the control group under the treatment of 2 μmol/L MPPa or single irradiation. However, cell viability decreased obviously under the treatment of high-dose MPPa (4 μmol/L), so 2 μmol/L MPPa was used for our experiments. Cell viability was related to the irradiation dose, which decreased with increasing laser irradiation (Fig. 1b). ROS generation was detected after MPPa-PDT and was notably increased in the MPPa-PDT group compared to the control group. The ROS levels in the MPPa, laser and NAC + MPPa-PDT (pretreated with NAC before irradiation) groups were far below those of the MPPa-PDT group (Fig. 1c).

### MPPa-PDT suppresses MCF-7 cell migration, invasion and MMP-9 expression

**Fig. 2:**
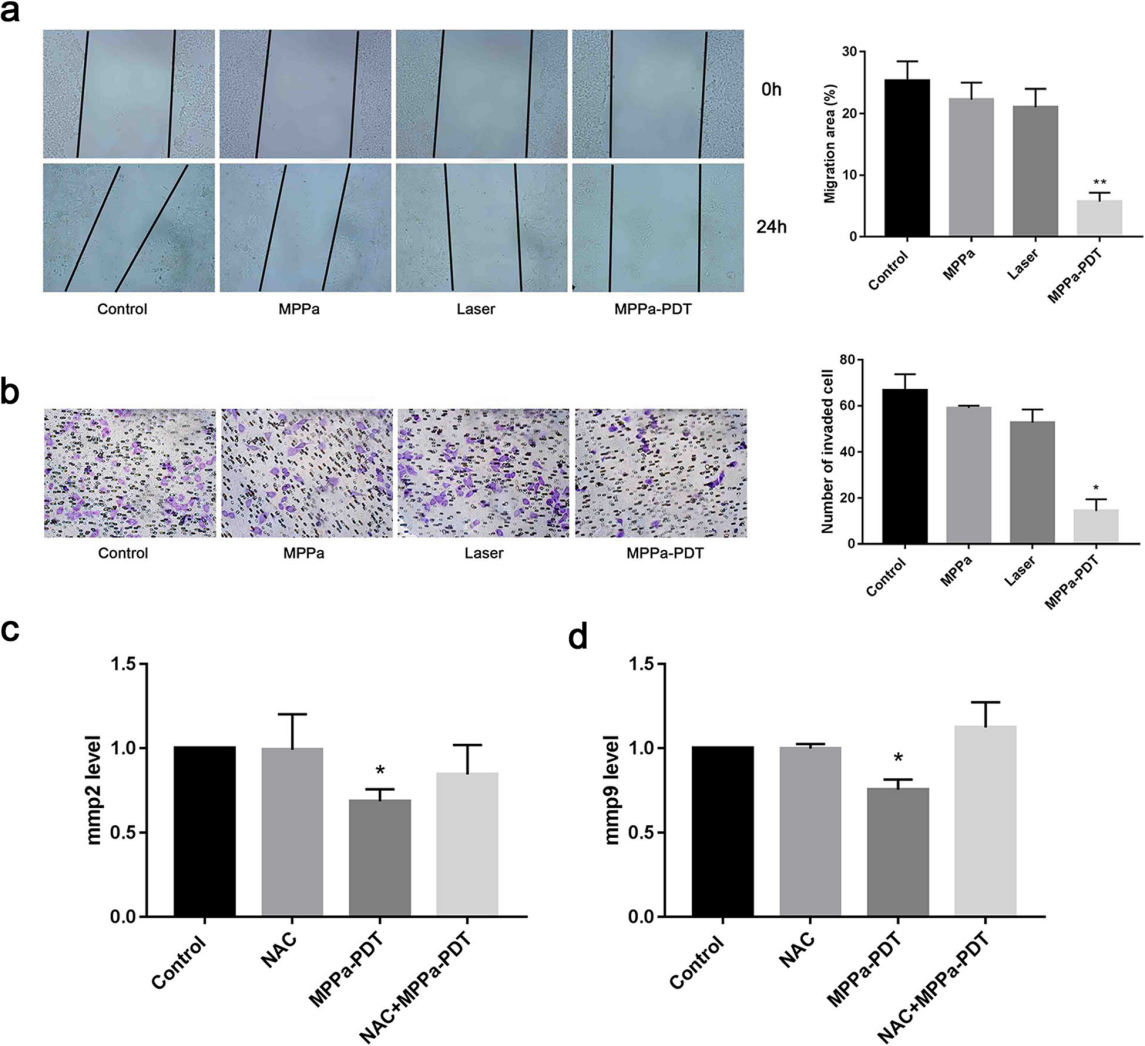
MPPa-PDT inhibits the migration and invasion of MCF-7 cells. **a** The effect of MPPa-PDT on migration in MCF-7 cells was detected by wound healing assay after 24 h (magnification, × 100). **b** The effect of MPPa-PDT on invasion in MCF-7 cells was measured by Matrigel invasion assay after 48 h (magnification, × 200). **c** The expression of MMP2 was measured by RT-PCR after 24 h. **d** The expression of MMP9 was measured by RT-PCR after 24 h. (n = 3; *P < 0.05 versus control group, **P < 0.01 versus control group)

Data from the wound healing assay and invasion assay indicated that treatment with MPPa-PDT effectively suppressed the migration (Fig. 2a) and invasion of MCF-7 cells (Fig. 2b) compared to the three control cells (control group, MPPa group and laser group). The expression of MMP-2 and MMP-9 was also significantly downregulated by MPPa-PDT (Fig. 2c and d).

### MPPa-PDT downregulates the PI3K/Akt/NF-κB signaling pathway

**Fig. 3:**
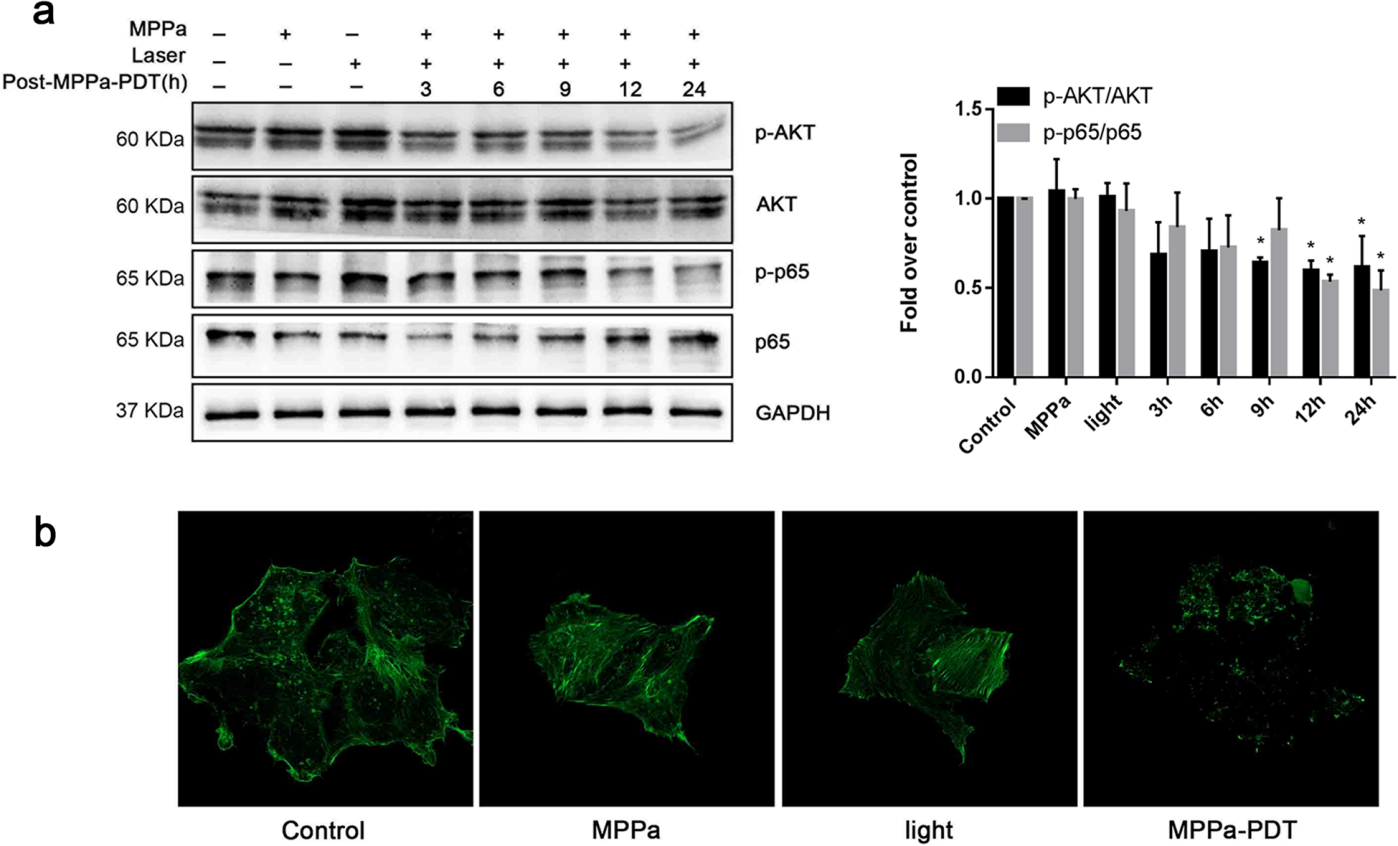
MPPa-PDT reduces the phosphorylation of Akt and NF-κB. **a** The expression levels of Akt, p-Akt, p65, and p-p65 were detected by western blotting. **b** Cytoskeleton was significantly reduced after MPPa-PDT, as detected by confocal microscopy (magnification, × 600). (n = 3; *P < 0.05 versus control group)

We collected the cells at different times (3, 6, 9, 12, 24 h) after PDT, and western blotting demonstrated that the protein expression of p-Akt and p-p65 was decreased following MPPa-PDT compared to the control groups (Fig. 3a). Then we measured the changes in the F-actin cytoskeleton in all four groups and found that MPPa-PDT significantly reduced the cytoskeleton (Fig. 3b).

### MPPa-PDT suppresses MCF-7 cell metastasis by inhibiting PI3K/Akt/NF-κB-dependent MMP-9 expression

**Fig. 4:**
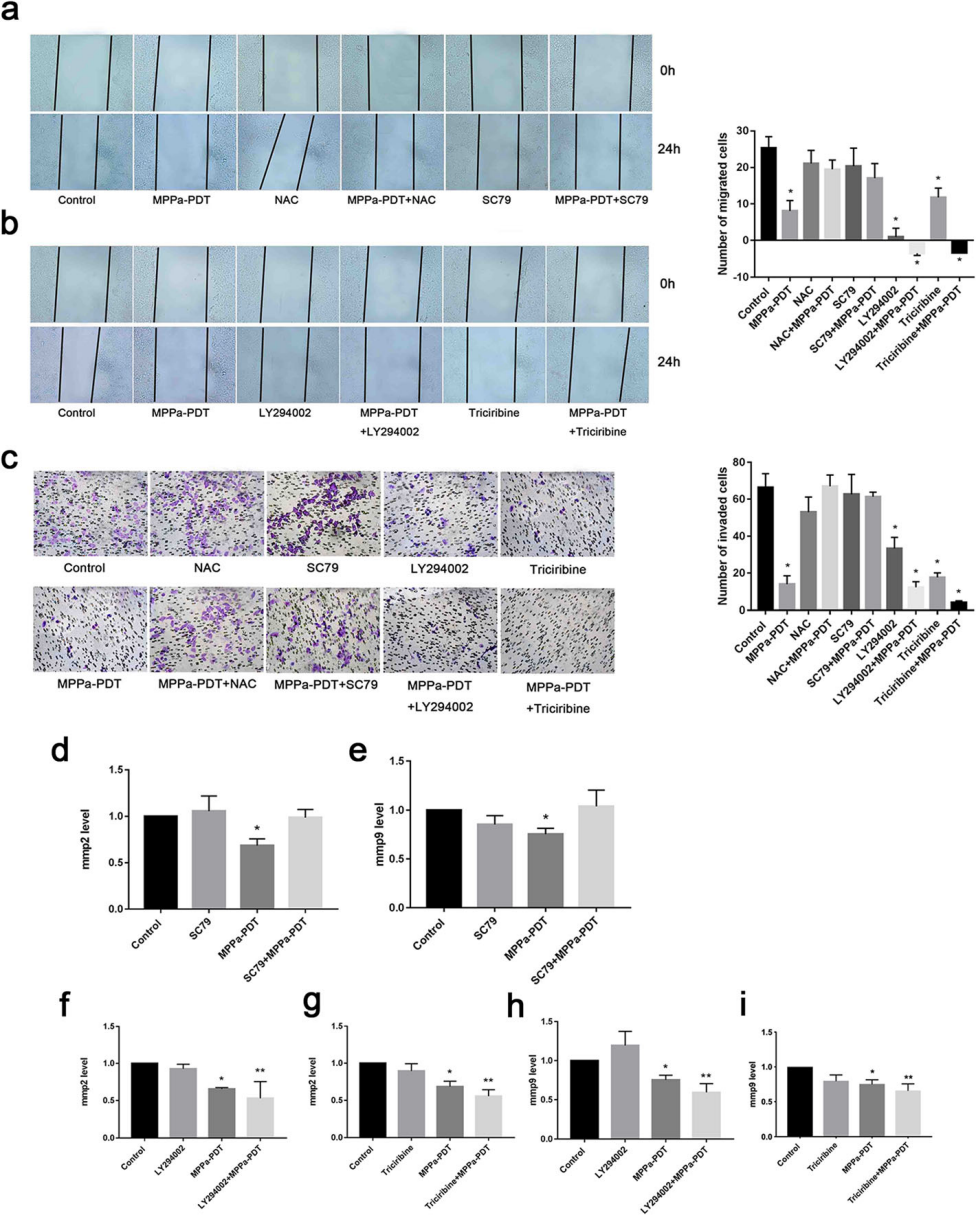
MPPa-PDT inhibits metastasis in MCF-7 cells through the Akt/NF-κB-dependent MMP9 signaling pathway. **a** The effect of NAC and SC79 on migration after MPPa-PDT (magnification, × 100). **b** The effect of LY294002 and triciribine on migration after MPPa-PDT (magnification, × 100). **c** The effect of NAC, SC79, LY294002 and triciribine on invasion after MPPa-PDT (magnification, × 200). **d** The effect of SC79 on MMP2 expression after MPPa-PDT. **e** The effect of SC79 on MMP9 expression after MPPa-PDT. **f** The effect of LY294002 on MMP2 expression after MPPa-PDT. **g** The effect of triciribine on MMP2 expression after MPPa-PDT. **h** The effect of LY294002 on MMP9 expression after MPPa-PDT. **i** The effect of triciribine on MMP9 expression after MPPa-PDT. (*n* = 3; *P < 0.05 versus control group, **P < 0.01 versus control group)

To investigate the underlying mechanism of action of MPPa-PDT on MCF-7 cell metastasis, MCF-7 cells were pretreated with inhibitors of PI3K (LY294002) and Akt (triciribine), an activator of Akt (SC79) and a scavenger of ROS (NAC) for 12 h before irradiation. Elimination of ROS and activation of Akt abolished the effects of MPPa-PDT on migration (Fig. 4a), while PI3K inhibitor LY294002 and Akt inhibitor triciribine enhanced these effects (Fig. 4b). The changes in cell invasion had the same tendency (Fig. 4). Significant increases in the expression levels of MMP-2 and MMP-9 were observed after pretreatment of cells with Akt activator SC79 for 12 h (Fig. 4d and e). Suppression of PI3K and Akt further reduced MMP-2/MMP-9 gene expression (Fig. 4f-i).

### MPPa-PDT induces MCF-7 cell cytoskeleton destruction by inhibiting PI3K/Akt/NF-κB

**Fig. 5:**
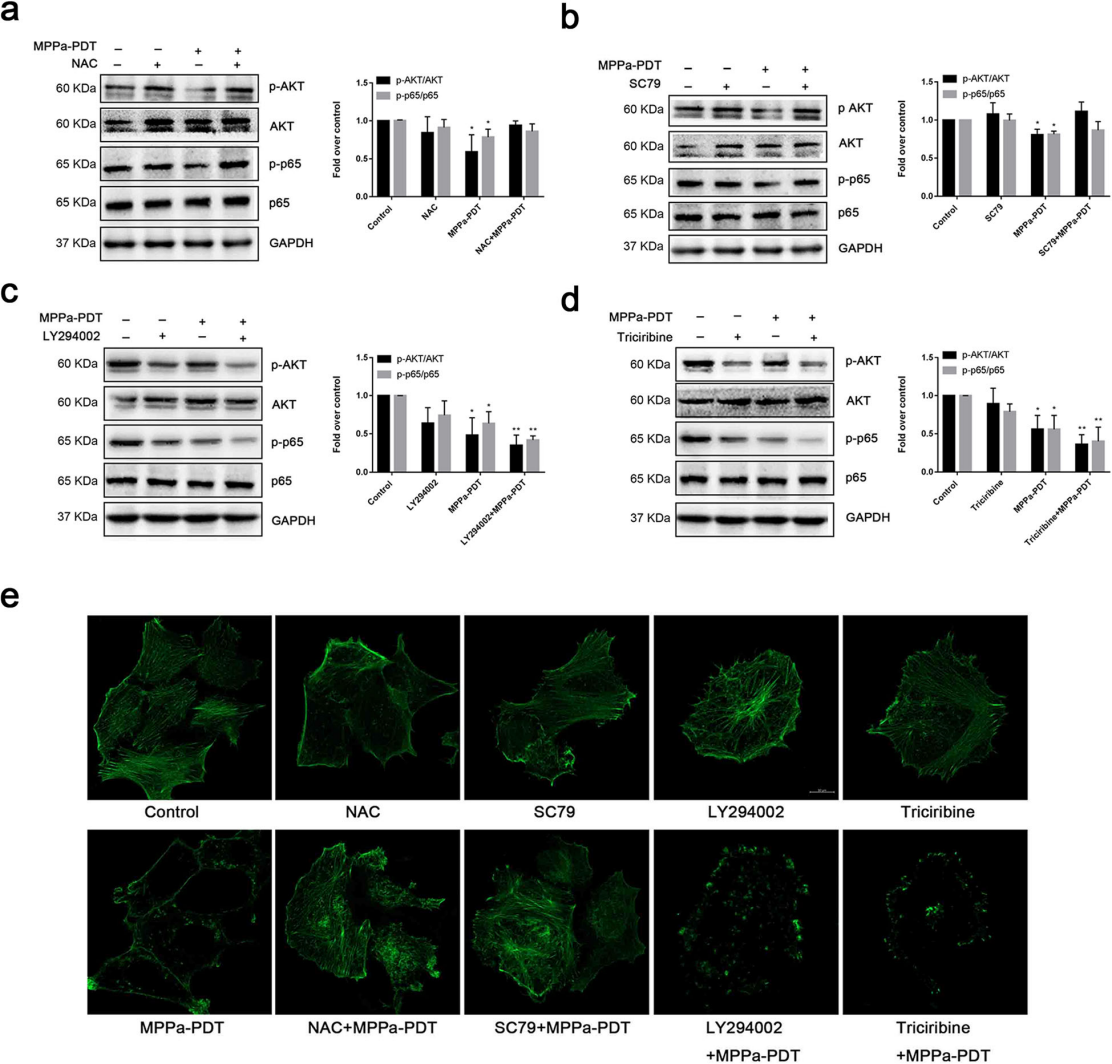
MPPa-PDT inhibits metastasis in MCF-7 cells through the Akt/NF-κB signaling pathway. **a** The effect of NAC on the expression levels of Akt, p-Akt, p65, and p-p65 after MPPa-PDT. Quantifications of the proteins are shown. **b** The effect of SC79 on the expression levels of Akt, p-Akt, p65, and p-p65 after MPPa-PDT. Quantifications of the proteins are shown. **c** The effect of LY294002 on the expression levels of Akt, p-Akt, p65, and p-p65 after MPPa-PDT. Quantifications of the proteins are shown. **d** The effect of triciribine on the expression levels of Akt, p-Akt, p65, and p-p65 after MPPa-PDT. Quantifications of the proteins are shown. **e** The effect of NAC, SC79, LY294002 and triciribine on the F-actin cytoskeleton after MPPa-PDT (magnification, × 600). (n = 3; *P < 0.05 versus control group, **P < 0.01 versus control group)

Phosphorylation of Akt and p65 was upregulated after application of the ROS scavenger NAC (Fig. 5a) and Akt activator SC79 (Fig. 5b). Additionally, the application of the PI3K inhibitors LY294002 (Fig. 5c) and the Akt inhibitor triciribine (Fig. 5d) clearly decreased the levels of p-Akt and p-p65. MPPa-PDT significantly reduced the cytoskeleton compared with the control groups, and inhibition of PI3K or Akt further aggravated this effect. Elimination of ROS and activation of Akt alleviated this effect of MPPa-PDT (Fig. 5e).

### MPPa-PDT decreases the tumor progression of MCF-7 cells in vivo

**Fig. 6:**
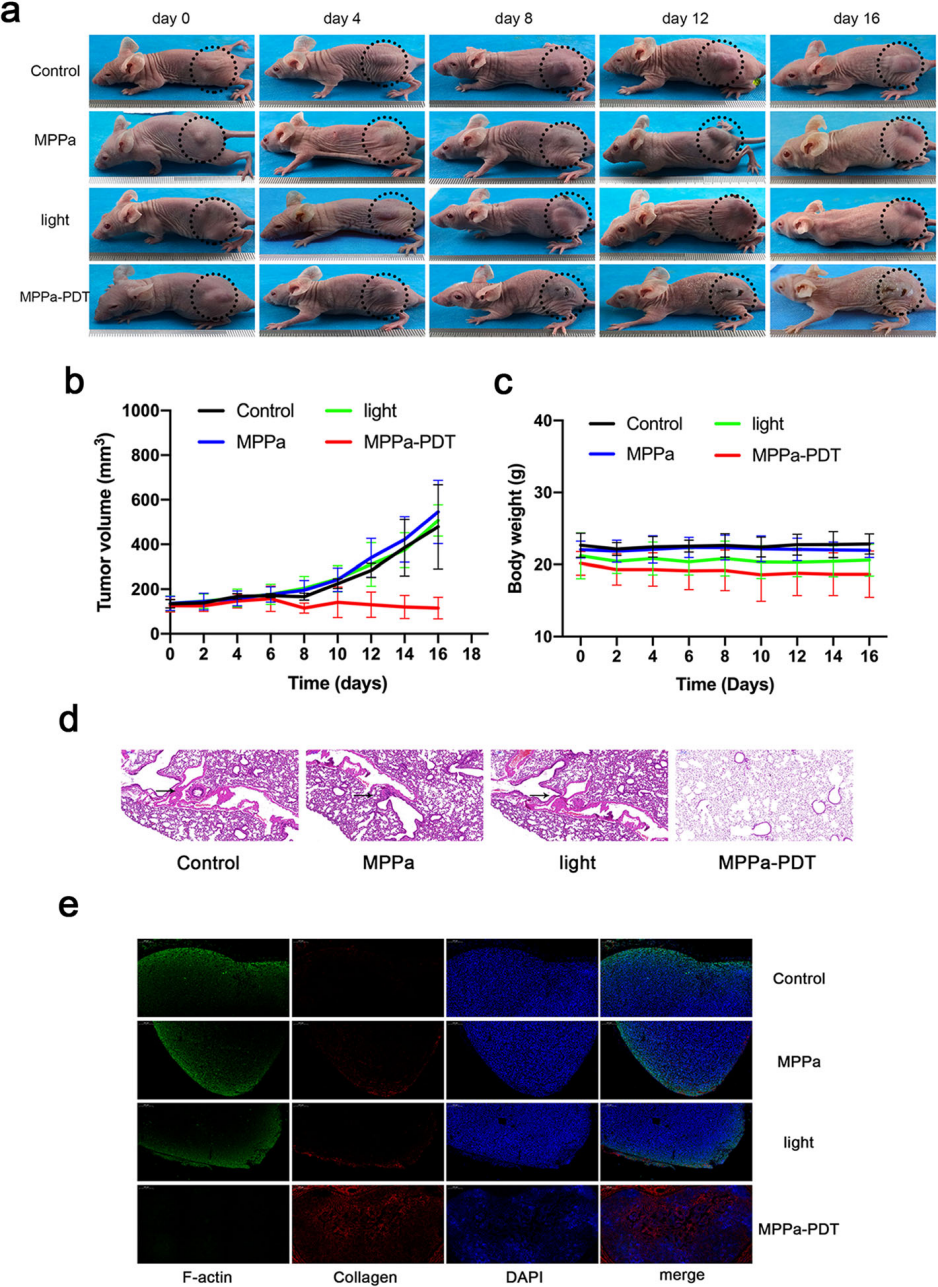
MPPa-PDT inhibits tumor metastasis in vivo. **a** Representative photos of the tumor-bearing mice in the four groups. **b** Tumor volume changes of the four groups over the course of treatments. **c** Body weight changes of the four groups over the course of treatments. **d** Representative H&E staining images of lung tissues from the mice in the four groups. **e** Collagen and cytoskeleton of tumor tissues were measured by immunofluorescence staining (scale bar, 200 μm)

The conditions of tumors were recorded for 16 d after different treatments, and photographs of mice were taken every 2 days to reveal the changes in tumors (Fig. 6a). Tumors in these control groups grew rapidly, suggesting that MPPa-only or laser-only had little therapeutic effect on the tumor-bearing mice. The tumor volumes in the MPPa-PDT group were visibly smaller than those in the control groups, indicating that the MCF-7 xenograft was sensitive to MPPa-PDT (Fig. 6b). During the treatment period, the weights of all the tumor-bearing mice showed no noticeable changes, which implies that MPPa has little systemic toxicity in mice (Fig. 6c).

### MPPa-PDT decreases the tumor metastasis of MCF-7 cells in vivo

Lung tissues were harvested to perform the hematoxylin and eosin (H&E) histopathological analysis. In the three control groups, there was apparent generation of micrometastatic foci; in contrast, there were few metastases in the MPPa-PDT group (Fig. 6d). Above mentioned results demonstrated that MPPa-PDT possessed successful therapeutic efficacy in inhibiting tumor metastasis. We analyzed the F-actin cytoskeleton and collagen of tumor tissues through immunofluorescence assay. The cytoskeleton in the MPPa-PDT group exhibited undoubted destruction, while the three control groups exhibited relatively complete structures. Collagen in the three control groups was destroyed, while in MPPa-PDT, it was apparent (Fig. 6f). There was no significant difference among the three control groups.

## Discussion

Due to the limitations of current clinical treatment of breast cancer and accompanying unsatisfied prognosis, safe and effective therapy methods are urgently needed [24]. PDT exhibits unique advantages, such as minimal invasion to the human body, little system toxicity and no resistance [25]. PS, as an important element of PDT, should be readily available, inexpensive, and stable and possess an absorption peak at longer wavelengths. MPPa is a desirable photosensitizer that possesses good stability and a 630 nm absorbance wavelength [23]; thus, it was chosen for the present study.

ROS play the most pivotal role in PDT [26], which is implicated in cellular homeostasis and possesses bidirectional effects on cellular processes. Chemotherapeutic drugs exert anticancer effects via the generation of ROS such as doxorubicin, cisplatin and mitomycin C in many cancer diseases. Moderate ROS levels influence inflammation of tissues or cells, cellular defense systems, and progression of cancer. Overproduction of ROS induces cancer cell apoptosis; nucleic acid, lipid, and protein damage; and suppression of pro-survival pathways [27]. Therefore, appropriate levels of ROS may be an effective therapeutic method. Our previous study showed that ROS produced by PDT-induced breast cancer cell apoptosis, including MCF-7 and MDA-MB-231 cells. Considering that cancer metastasis is the most important cause of death in breast cancer patients [23], we wondered whether MPPa-PDT could influence breast cancer cell line metastasis and what the interrelated mechanisms would be. Our study found that ROS were significantly increased after MPPa-PDT treatment, which was compatible with previous studies.

High migration and invasion capability are major malignant characteristics of cancer cells, while ECM is the crucial barrier to cancer metastasis. Metastasis occurs when cancer cells spread to other organs from a primary tumor site and form new tumors [28, 29], including migration and invasion, intravasation, arrest of cancer cells and extravasation, as well as metastatic colonization [29]. Metastases are a leading cause of breast cancer mortality [30], and suppression of metastasis would ameliorate patient prognosis. Our study found that MPPa-PDT alleviated the invasion and migration of MCF-7 cells, suppressed the expression of MMP-2 and MMP-9, and inhibited Akt and NF-κB phosphorylation.

MMP-9 is an irreplaceable player in the degradation of ECM [31] whose abnormal expression disequilibrates ECM and contributes to cancer cell metastasis [32]. The expression of matrix metalloproteinase-9 is increased in invasive breast cancer, and the cause of this phenomenon is related to the mutual regulation of various intracellular signal transduction factors under different stimulation [33]. MMP-9 transcription is influenced by two major transcriptional factors, NF-κB and AP-1. The most common signals upstream of these two molecules are PI3K/Akt and MAPK [34, 35], which manage survival, metastasis and drug resistance [36, 37]. Inhibitors of the PI3K/Akt signaling pathway have been clinically applied for cancer treatment [38]. The present study showed that MPPa-PDT reduced MMP-9 and MMP-2 transcription and that MPPa-PDT also suppressed Akt phosphorylation and NF-κB activation. We used pharmacologic inhibitors of ROS (NAC), PI3K (LY294002), and Akt (triciribine) and an activator of Akt (SC79) to identify the intrinsic mechanism. Inhibition of PI3K and Akt further reduced Akt and NF-κB protein phosphorylation and regulated downstream signaling molecules, which in turn resulted in a reduction in MMP2 and MMP9 gene expression. At the same time, the capacities of migration and invasion of MCF-7 were subdued. However, suppression of ROS and activation of Akt specifically diminished MPPa-PDT’s effect on the expression of Akt and NF-κB proteins and the MMP-9 and MMP-2 genes, as well as cell metastatic ability. These results suggest that MPPa-PDT inhibited MCF-7 migration and invasion through the Akt/NF-κB/MMP-9/MMP-2 signaling axis by upregulating ROS production.

The F-actin cytoskeleton governs numerous biological functions, including cell contraction, cell motility and vesicle trafficking, with a well-configured network [39]. We investigated the changes in F-actin because it is known to regulate tumor cell invasion and migration [40, 41]. The results showed that MPPa-PDT destroyed the cytoskeleton, in contrast with the three control groups. Additionally, apparent disorganization of the cytoskeleton was discovered after administration of the PI3K inhibitor LY294002 and the Akt inhibitor triciribine. The cells had well-defined actin filaments pretreated with NAC or SC79. These results demonstrated that MPPa-PDT inhibited MCF-7 migration and invasion through the Akt/NF-κB signaling pathway via ROS.

To further examine the effects of MPPa-PDT on tumor growth and metastasis in vivo, MCF-7 cells were xenografted to nude mice. During the treatment period, tumors in the three control groups grew rapidly, while those in the MPPa-PDT group were obviously inhibited, indicating that MPPa-PDT can inhibit tumor growth. The major lung tissue was collected and subjected to H&E staining. There were obvious lung micrometastatic foci in the three control groups but no significant micrometastatic foci in the MPPa-PDT group, indicating that MPPa-PDT can inhibit tumor metastasis.

Cytoskeletal F-actin and collagen were used to evaluate the destruction of the ECM in the present study. The results showed that collagen deposition in the three control groups decreased obviously, while the collagen in the MPPa-PDT group exhibited no significant change. ECM destruction is involved in metastasis, and the primary component of ECM is collagen; thus, the integrity of collagen can frustrate the metastatic capacity of tumors. According to the F-actin staining, the network structure of F-actin was depolymerized significantly after MPPa-PDT treatment; conversely, the network structure of F-actin was notably visible in the other groups. These results showed that the tumor metastasis capacity in the MPPa-PDT group was weaker than that in the other groups. These results also demonstrated that MPPa-PDT can inhibit tumor metastasis.

## Conclusions

The present study suggests that MPPa-PDT inhibited the migration and invasion of breast cancer cells through the Akt/NF-κB-dependent MMP-9 signaling pathway by upregulating ROS production. Downregulation of MMP-2 and MMP-9 expression induced by MPPa-PDT may enhance the therapeutic efficacy for breast cancer by restricting metastasis by upregulating cell apoptosis and necrosis. These findings suggest that MPPa-PDT is a promising therapeutic method for breast cancer.

## Data Availability

The datasets used and/or analyzed during the current study are available from the corresponding author on reasonable request.

## Abbreviations

Akt: Protein kinase B
CCK-8: Cell viability and cytotoxicity test kit
DMEM: Dulbecco’s modified Eagle’s medium
ECM: Extracellular matrix
H&E: Hematoxylin and Eosin
MMPs: Matrix metalloproteinases
MPPa: Pyropheophorbide-α methyl ester
OD: Optical densities
PCR: Polymerase-chain reaction
PDT: Photodynamic therapy
PFA: Paraformaldehyde
PI3K: Phosphoinositide 3-kinase
ROS: Reactive oxygen species
TV: Tumor Volume
